# Magnetic Field Effects Induced in Electrical Devices Based on Cotton Fiber Composites, Carbonyl Iron Microparticles and Barium Titanate Nanoparticles

**DOI:** 10.3390/nano12050888

**Published:** 2022-03-07

**Authors:** Gabriel Pascu, Octavian Madalin Bunoiu, Ioan Bica

**Affiliations:** 1 Faculty of Physics, West University of Timisoara, 4 V. Parvan Avenue, 300223 Timisoara, Romania; gabriel.pascu@e-uvt.ro (G.P.); ioanbica50@gmail.com (I.B.); 2 Institute of Advanced Environmental Research, West University of Timisoara, 4 V. Parvan Avenue, 300223 Timisoara, Romania

**Keywords:** barium titanate nanoparticles, carbonyl iron, cotton fabric, magnetopiezoelectric effects, magnetocapacitive effects, magnetoresistive effects

## Abstract

This work consists in the process of preparing magnetic active composite materials based on cotton fibers, iron carbonyl microparticles and barium titanate nanoparticles, and the electrical devices manufactured with them. For different compositions of the aforementioned ingredients, three such composites are manufactured and compacted at constant pressure between two electrodes. In the absence and in the presence of a magnetic field, using an RLC bridge, magnetocapacitive, magnetoresistive and magnetopiezoelectric effects are highlighted in the custom fabricated devices. It is shown that these effects are significantly influenced by the composition of the materials. Based on the model elaborated in this paper, the mechanisms that contribute to the observed effects are described and the theoretical predictions are shown to agree with the experimental data. The obtained results can be used in the assembly of hybrid magnetic active composites, which are low cost, ecological and have other useful physical characteristics for applications.

## 1. Introduction

Magnetic liquids (MLs) [1,2,3,4,5,6,7,8,9,10,11], magnetorheological suspensions (MRSs) [12,13,14,15,16,17,18,19,20,21,22,23,24,25,26,27,28] and magnetorheological elastomers (MREs) [29,30,31,32,33,34,35,36,37,38,39,40,41,42,43] are characterized by the fact that they possess a magnetizable phase (ferri-ferromagnetic particles) and additives (fibers of natural and/or artificial polymers, nanotubes, graphene nanopallets, etc.) undissolved in the base matrix, which can be either liquid in the case of MLs and MRSs or typically a silicone rubber in the case of MREs. Each component of the MLs, MRSs and MREs has different physical characteristics, but together, as a whole, they form magnetically active composite materials (MACs), with different physical characteristics compared to those of the constituent components.

When applying a magnetic field, the thermodynamic and transport characteristics of MACs change drastically, a useful property in medical diagnoses and therapies [4,5,6,7,8,9], vibration dampers and seismic shocks [20,43], medical devices [21,22,24], magnetic field sensors and mechanical deformations [23,25,37,38]. In order to achieve high-performance devices with MACs, the scientific community is preoccupied with improving their physical characteristics by finding new ways of preparation and characterization [1,2,3,6,7,12,13,14,15,16,17,18,19,23,25,27,28,29,30,31,32,33,34,35,39,40,41,42]. In these circumstances, we consider that research towards the assembly of MACs, using a magnetizable phase and additives in the form of nano/microparticles, would generate composite materials with physical characteristics suitable for various applications.

Following this research path, MACs based on cotton fibers (CF), carbonyl iron microparticles (CI) and barium titanate nanoparticles (nBT) are prepared and characterized in the paper. The process used was that of compacting at constant pressure the CF, CI and nBT between the two copper electrodes of the electrical devices (EDs). The MACs obtained are differentiated by the values of the volume fractions of CF, CI and nBT.

Using an RLC bridge, the equivalent electric capacity SCS, equivalent electric resistance *R* and electric voltage *U* are measured in the absence and in the presence of the magnetic field. From the evaluation of the obtained data, it is observed that in the EDs, the magnetic field induces magnetocapacitive ($\mu_{C}$), magnetoresistive ($\mu_{R}$) and magnetopiezoelectric ($\mu_{U}$) effects, which are significantly influenced by the volume fractions of the CF, CI and nBT components. The installation of these effects and their magnetic control leads us to the conclusion that these MACs are materials with distinct properties from MLs, MRSs and MREs. The model developed in the paper describes the physical mechanisms that contribute to the emergence of the obtained results and shows the agreement between the theoretical description and the experimental data. The effects make the use of MACs possible in detecting mechanical and acoustic vibrations and as sensors for deformations and mechanical stresses.

## 2. Materials and Methods

### 2.1. Manufacturing the Magnetic Active Composites (MACs)

**Figure 1:**
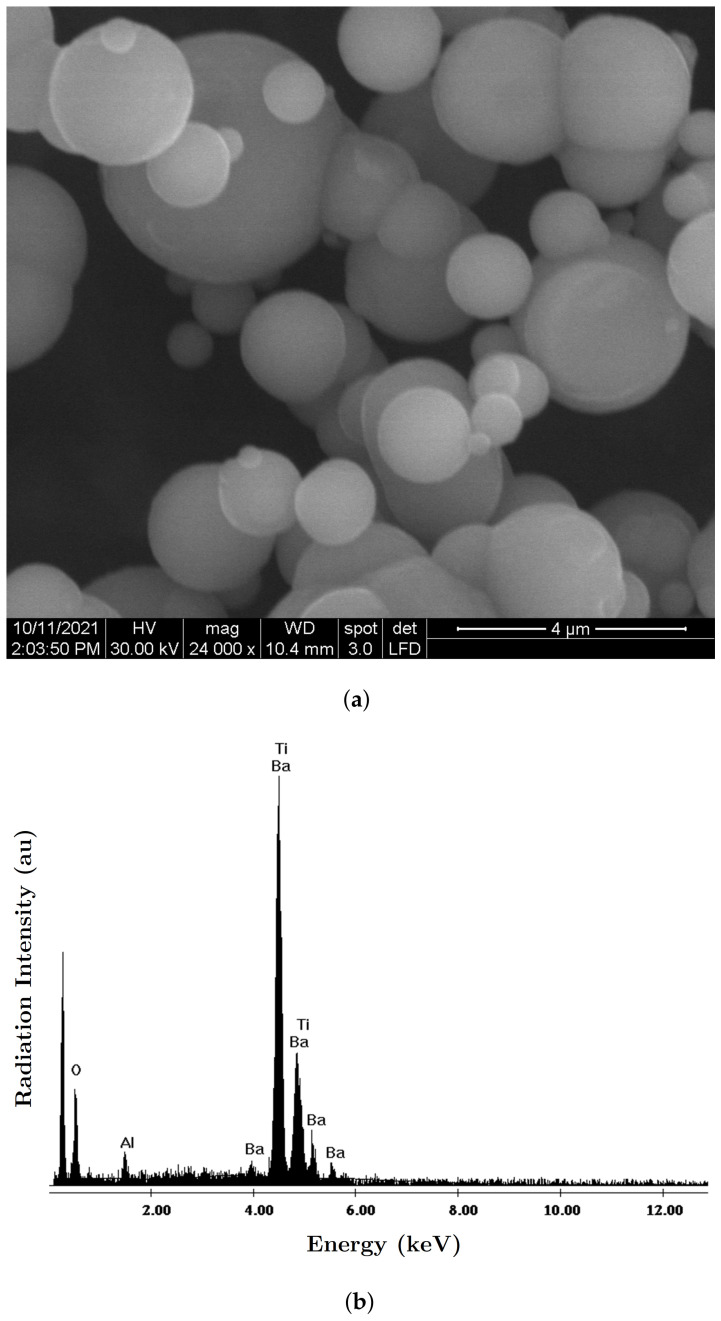
(**a**) SEM morphology of the nBT nanoparticles; (**b**) EDX spectra for the elemental analysis of nBT nanoparticles.

**Figure 2:**
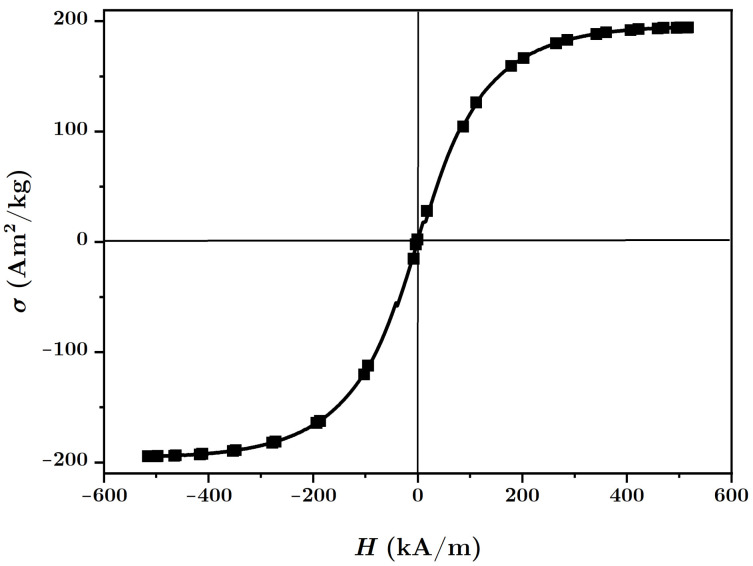
Magnetization curve of the CI microparticles.

**Figure 3:**
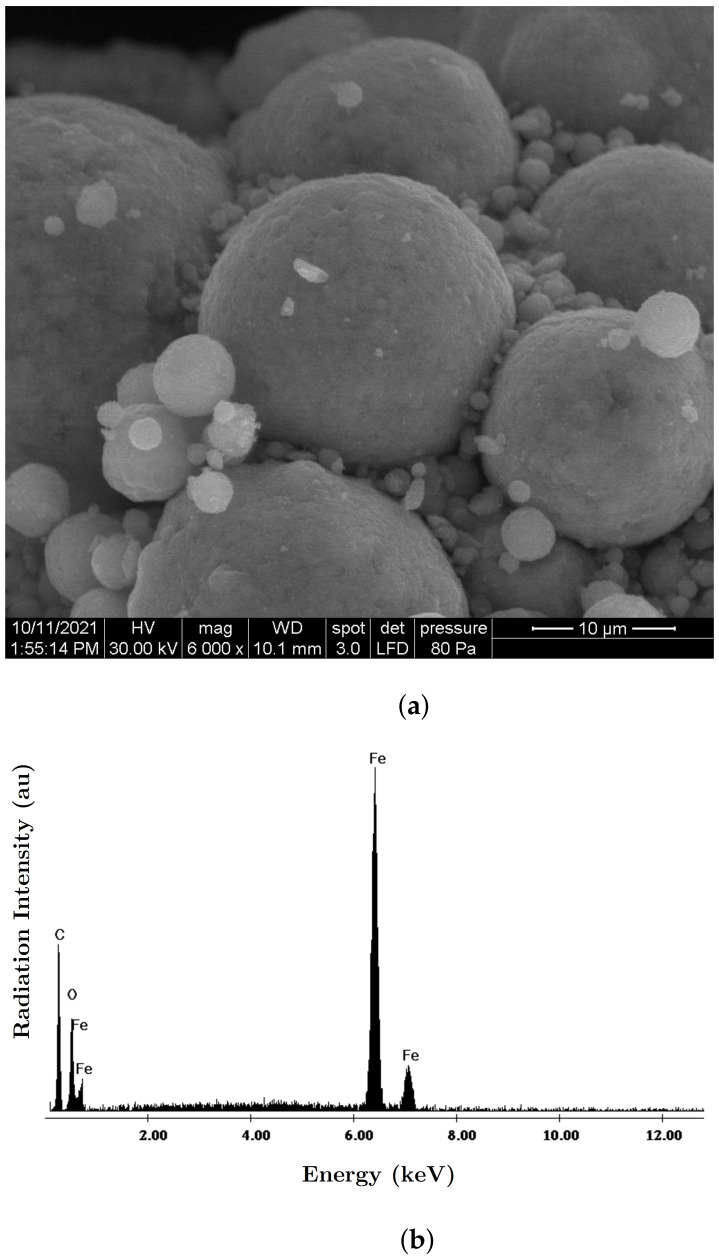
(**a**) SEM morphology of the CI microparticles; (**b**) EDX spectra for the elemental analysis of CI microparticles.

The necessary materials for manufacturing the MACs were:

(a) Barium titanate nanoparticles (nBT), from Sigma-Aldrich Chemie GmbH (Taufkirchen, Germany) with a maximum diameter of 100nm, purity of at least 97%, density $\rho_{\mathrm{nBT}}=6.08\;{g/cm}^{3}$, Curie point $t_{c\;\mathrm{nBT}}=130{\;}^{{}^{\circ}}$C, piezoelectric coefficient $d_{33}=85.6\;pC/N$ and relative dielectric permittivity $\epsilon_{r\;\mathrm{nBT}}=150$;
  The morphology of the nBT nanoparticles and their chemical analysis were highlighted using an Inspect S PANalytical instrument, coupled with an energy dispersive X-ray analysis detector (EDX), as shown in Figure 1.
(b) Carbonyl iron microparticles (CI), from Sigma-Aldrich Chemie GmbH (Taufkirchen, Germany), code C-3518, with an average diameter of 5μm, purity of at least 97%, density $\rho_{\mathrm{CI}}=7.68\;{g/cm}^{3}$ at $25{\;}^{{}^{\circ}}C$, and a magnetization curve as in Figure 2, plotted using an installation of the type used in [44]. This curve possessed an almost null surface area for an intensity of the magnetic field of H=545kA/m and the specific saturation magnetization of the CI microparticles was $\sigma_{s}=196\;kA\cdot m^{2}/kg$;
  The morphology of the CI microparticles and their chemical analysis were highlighted using an Inspect S PANalytical instrument, coupled with an energy dispersive X-ray analysis detector (EDX), as shown in Figure 3.
(c) The cotton tissue was a type of gauze bandage from Shanghai International Trading Corp. GmbH (Hamburg, Germany), composed of 10 layers of superimposed cotton fibers, each layer made of cotton fibers with a thickness of 0.30mm interwoven in the form of square-shaped meshes with dimensions of $1.35\times 0.68\times 0.30\;{mm}^{3}$. Out of 4 layers of tissue, each having the dimensions $30\times 30\times 1.20\;{mm}^{3}$, a packet was made, which is called here the cotton fiber tissue (CF), having a surface area $S_{\mathrm{CF}}=9.00\;{cm}^{2}$. On the surface of the tissue, the number of free spaces (rectangles) delimited by the fibers can be identified visually as $N_{0}=540$, with a precision of ±1%. The volume of a single such space was $V_{0}=1.35\;mm\times 0.68\;mm\times 1.20\;mm=1.1016\;{mm}^{3}$, which means that the whole free space in the tissue was $V=N_{0}V_{0}=0.594864\;{cm}^{3}$, and the volume of the fibers in the CF was $V_{f}=V_{\mathrm{CF}}-V=0.485\;{cm}^{3}$.

**Figure 4:**
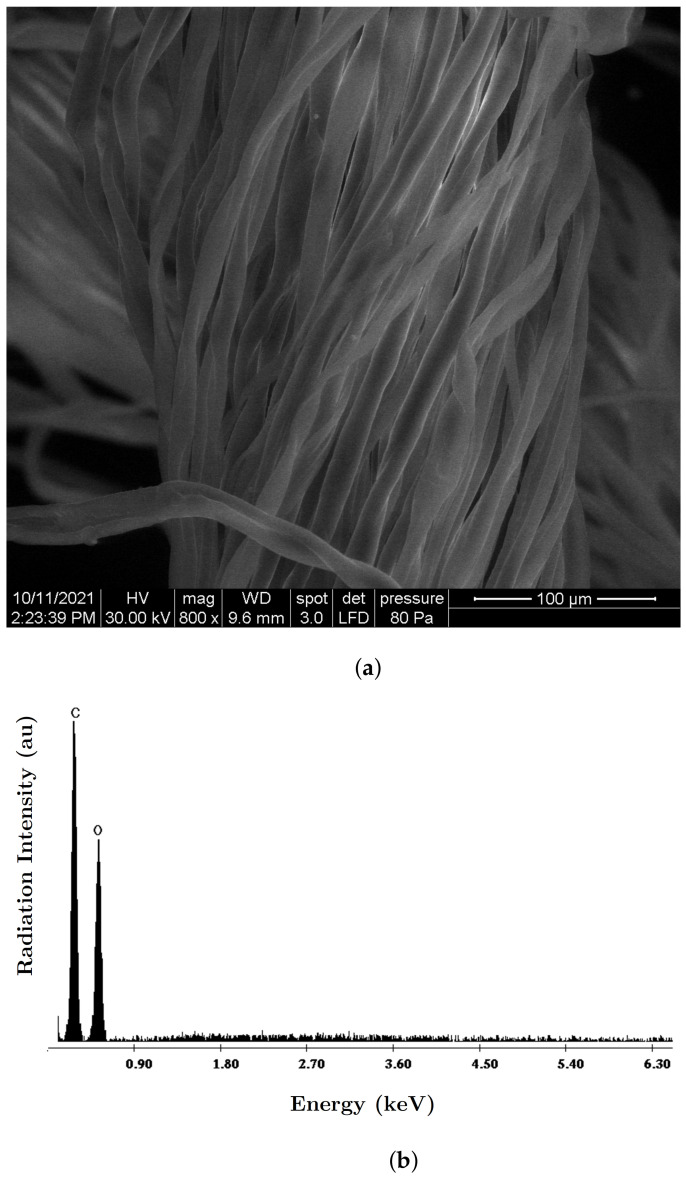
(**a**) SEM morphology of the CF microfibers; (**b**) EDX spectra for the elemental analysis of the CF.

The morphology of the fabric fibers and their chemical analysis were highlighted using an Inspect S PANalytical model coupled with an energy dispersive X-ray analysis detector (EDX) (Figure 4).

**Figure 5:**
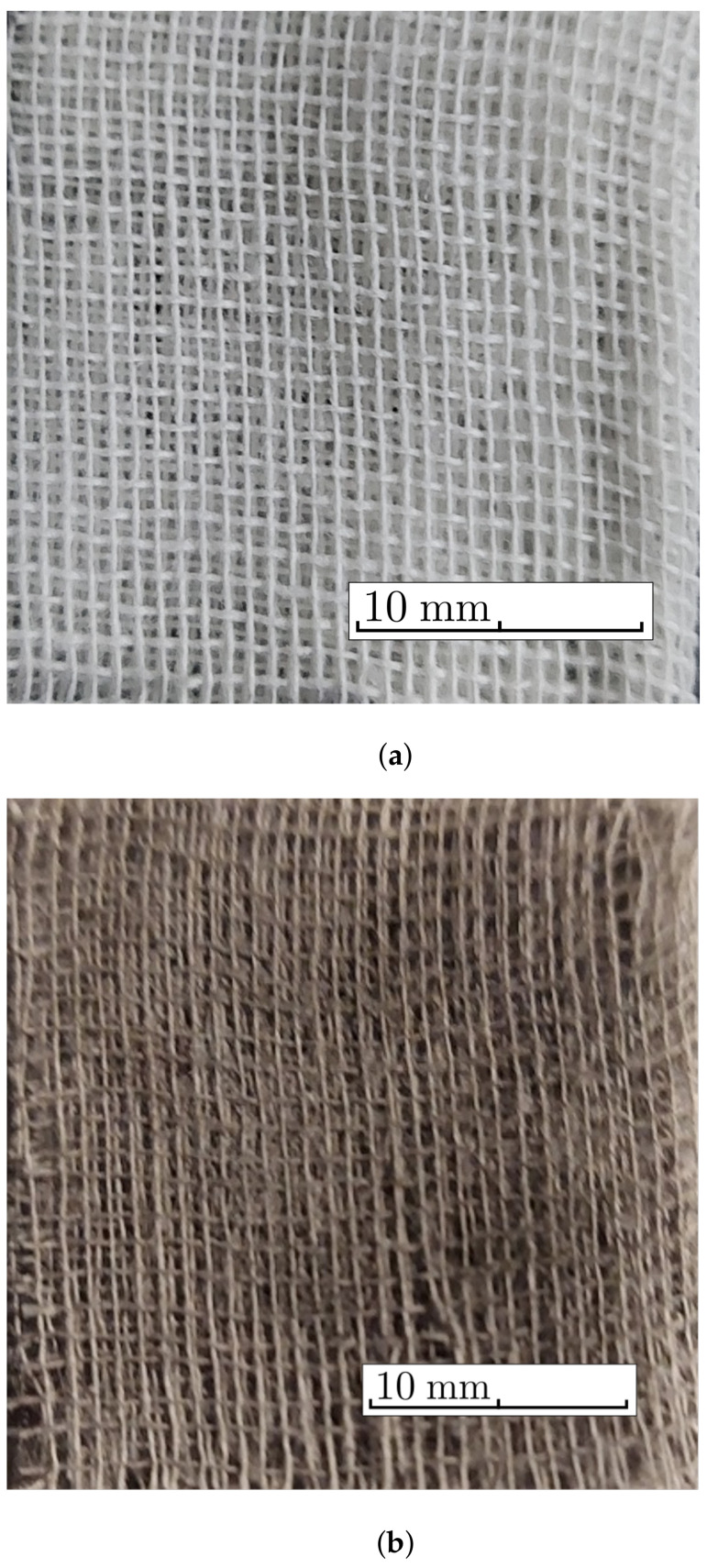
The cotton fabric CF: (**a**) before and (**b**) after doping with CI microparticles and nBT nanoparticles.

**Figure 6:**
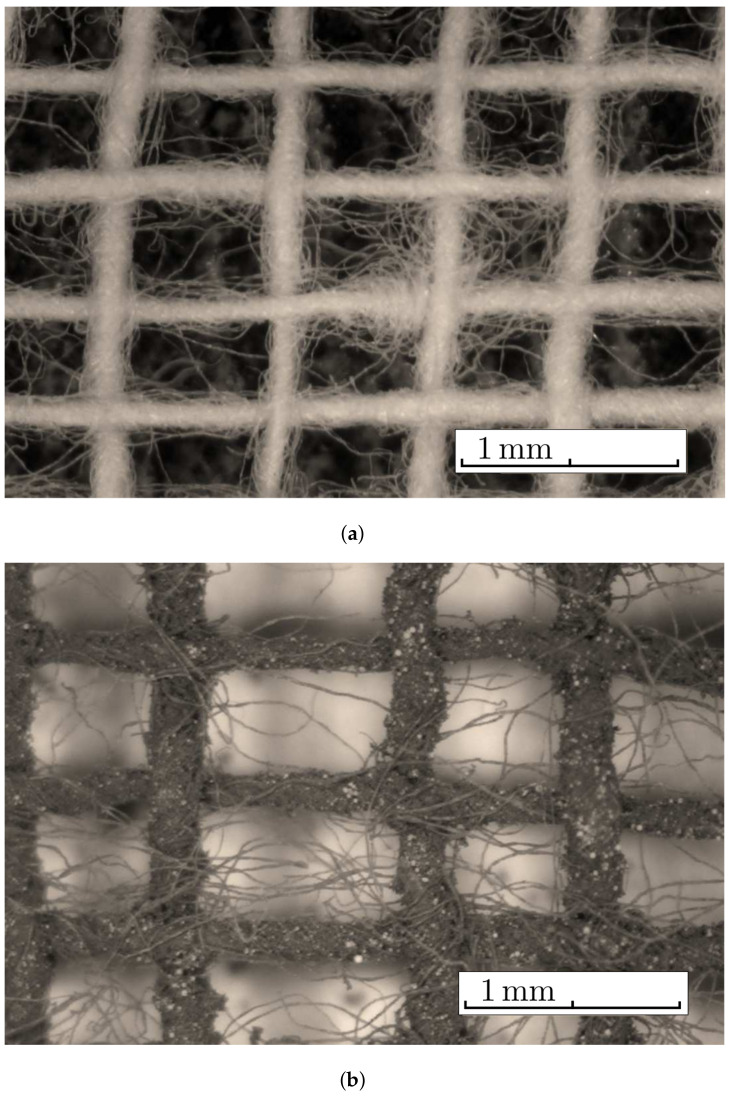
A layer of cotton fabric CF: (**a**) without particles; (**b**) doped with CI microparticles (black dots) and nBT nanoparticles (white dots). Photographs taken with a BPM-350 digital microscope for industrial inspection from Catchbest Technology Co., Ltd. (Beijing, China).

The steps for the manufacturing of the MACs were:

(1) Three distinct packets of CF were formed—CF${}_{1}$, CF${}_{2}$ and CF${}_{3}$—having a square geometry;
(2) Volumes $V_{\mathrm{CI}}$ of iron carbonyl microparticles, $V_{\mathrm{nBT}}$ of barium titanate nanoparticles were measured, in the quantities specified in Table 1;
(3) In each of the cotton fiber samples CF${}_{i}$ volumes $V_{\mathrm{CI}}$ of CI and $V_{\mathrm{nBT}}$ were inserted, and samples MAC${}_{i}$ were obtained (where i=1,2,3)—Figure 5 and Figure 6; It can be seen from Figure 4a that between the microfibers of the cotton yarn there were spaces in which the CI microparticles and the nBT nanoparticles can be retained.

**Figure 7:**
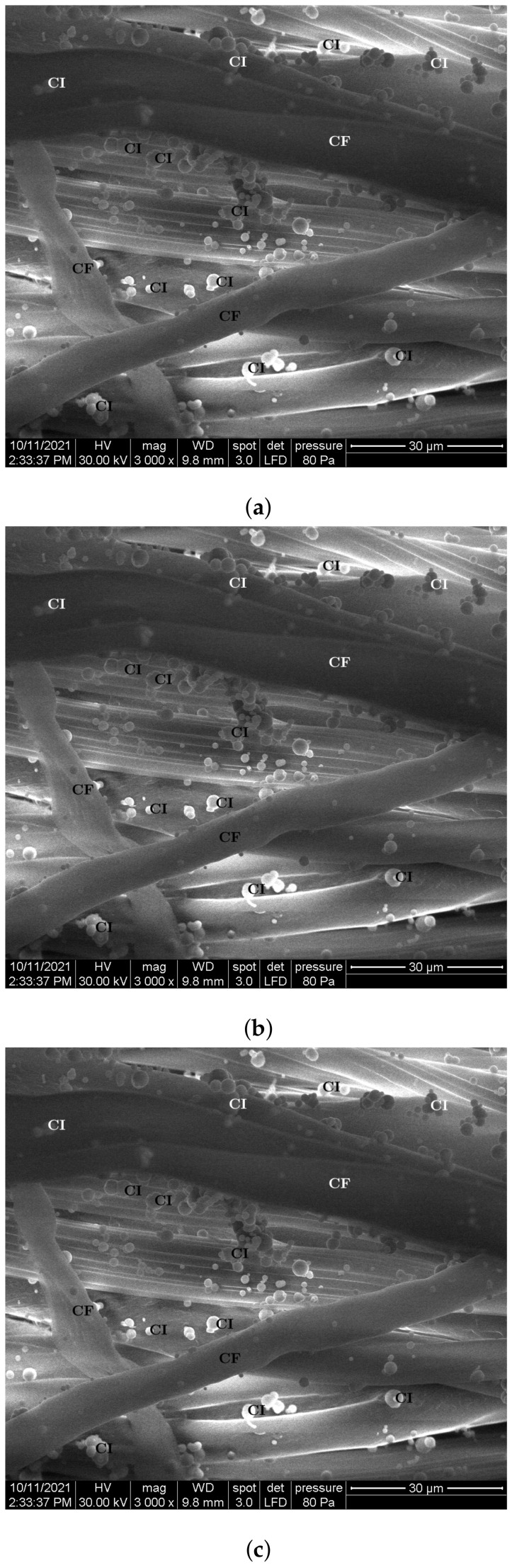
SEM morphology of samples: (**a**) MAC${}_{1}$; (**b**) MAC${}_{2}$; (**c**) MAC${}_{3}$; where: CF, cotton micorfibers; CI, carbonyl iron microparticles; nBT, barium titanate nanoparticles.

The morphology of the MAC samples were highlighted using the same Inspect S PANalytical instrument. Figure 7 shows that the particles used (carbonyl iron microparticles and barium titanate nanoparticles) are found between the microfibers of the cotton fabric threads and are well electrostatically anchored in them.

The volume of the MACs can be expressed as $V_{\mathrm{MAC}}=V_{\mathrm{CI}}+V_{\mathrm{nBT}}+V_{f}=S_{\mathrm{CF}}h_{0}$ where $S_{\mathrm{CF}}$ is the surface area of the MAC and $h_{0}$ its thickness. For $S_{\mathrm{CF}}=9.00\;{cm}^{2}$ and the values of the respective volumes, one can calculate (as shown in Table 1) the volume fractions Φ for the various components and the thickness $h_{0}$ of the MACs that have been compacted at a constant pressure p=7.98kPa.

**Figure 8:**
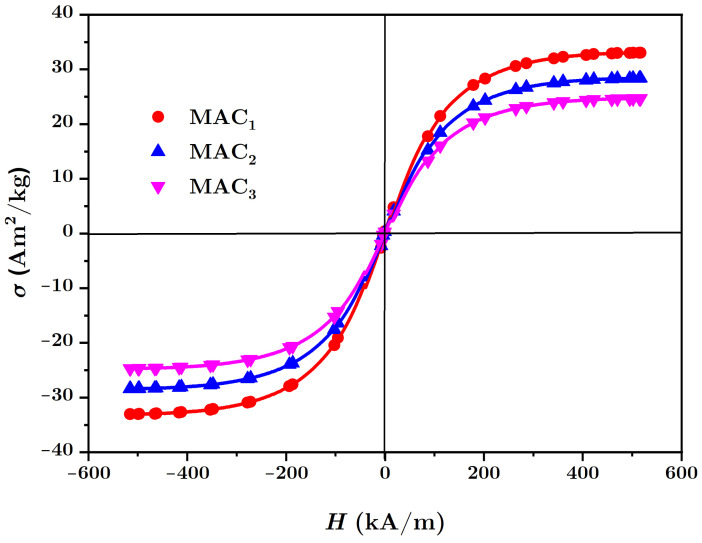
Magnetization curve of the magnetic active composites MACs.

Tracing the magnetization curve of the MACs membranes by using an installation as used in [44] is difficult to achieve. Here, we took into account the experimental fact according to which the existence of nonmagnetic additives has the effect of decreasing the relative saturation magnetization $\sigma_{s}$, but the allure of the magnetization curve stays the same with the increase in intensity of the external magnetic field *H*. It is known [45] that, between the relative saturation magnetization $\sigma_{s}$ of microparticles and the relative saturation magnetization $\sigma_{s_{i}}$ of the MACs, there is the relation $\mu_{0}\sigma_{s}=\Phi_{{\mathrm{CI}}_{i}}\mu_{0}\sigma_{s_{i}}$ for i=1,2,3, where $\mu_{0}$ is the magnetic permittivity of the vacuum and $\Phi_{{\mathrm{CI}}_{i}}$ is the volume fraction of the CI microparticles in the MACs (see Table 1). Based on these considerations, the magnetization curves of MACs are those shown in Figure 8, where it can be seen that the relative saturation magnetization of the CI microparticles is 5.90 times higher than that of ${MAC}_{1}$, 6.80 times higher than that of ${MAC}_{2}$ and 7.87 times higher than that of ${MAC}_{3}$.

### 2.2. Fabricating the Electrical Devices (EDs)

**Figure 9:**
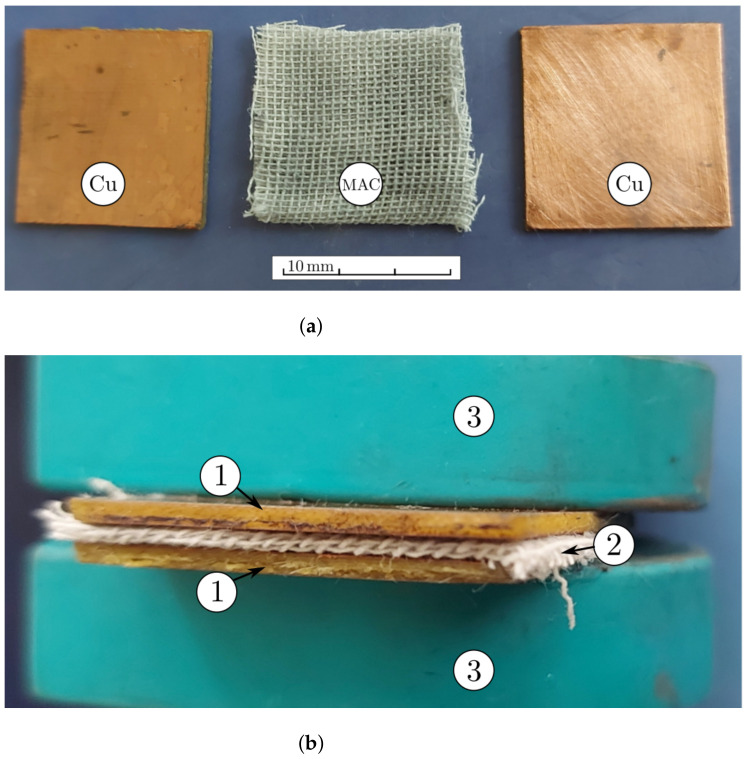
(**a**) Pair of Cu plates and MAC; (**b**) the device, while being pressed: 1, copper electrode; 2, MAC composite; 3, fixing elements.

For the manufacture of the EDs, copper coated plates (Cu) of type LMM 100x210E1 acquired from Electronic Light Tech SRL (Bucharest, Romania), having dimensions of 210mm×100mm×1mm were used, as shown in Figure 9. The plate itself is made of epoxy resin, of type FR4, and is reinforced with glass fiber. On one side of the plate, there is a 35μm thick electrolytic copper foil.

**Figure 10:**
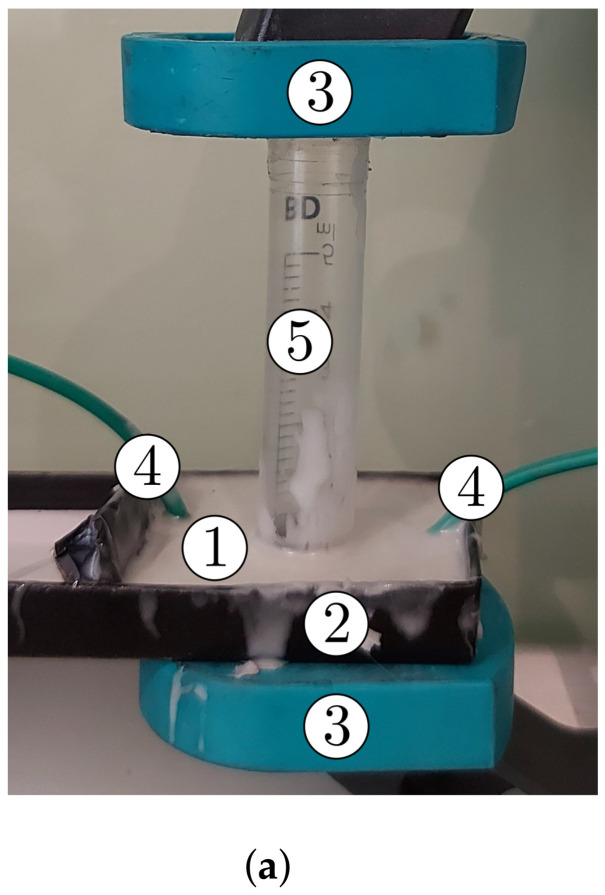

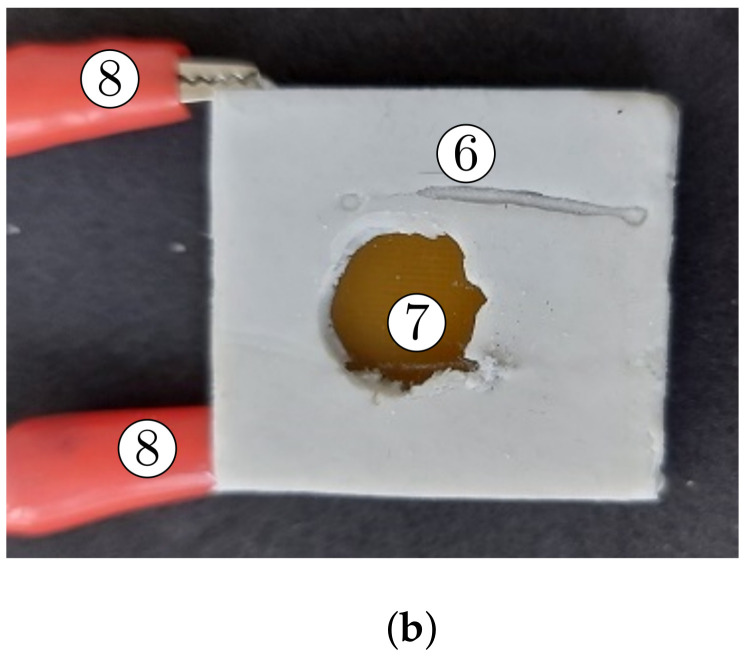
(**a**) Ensemble [46] featuring clamping press and mold, with the electrical device: 1, electrical device; 2, mold body; 3, clamping heads; 4, electrical conductors; 5, fixing cylinder assembly, consisting of copper boards with hybrid membrane; (**b**) electrical device overview: 6, silicone rubber sheath; 7, electrical device body; 8, electrical terminals.

The steps for the manufacturing of the EDs were:

(1) From the Cu plate, smaller plates of dimensions 30mm×30mm×1mm were cut and each of the two plates were paired in a packet, for a total of 3 packets;
(2) Between the electroconductive surface of each pair of plates an MAC was introduced (Figure 9a) and by pressing, (Figure 9b) the EDs were obtained;
(3) Liquid silicone rubber with catalyst was poured in order to contain the MAC components. It was poured as shown in Figure 10a. After about 24 h, the MACs coated in silicone rubber were obtained as shown in Figure 10b).

### 2.3. Experimental Installation

**Figure 11:**
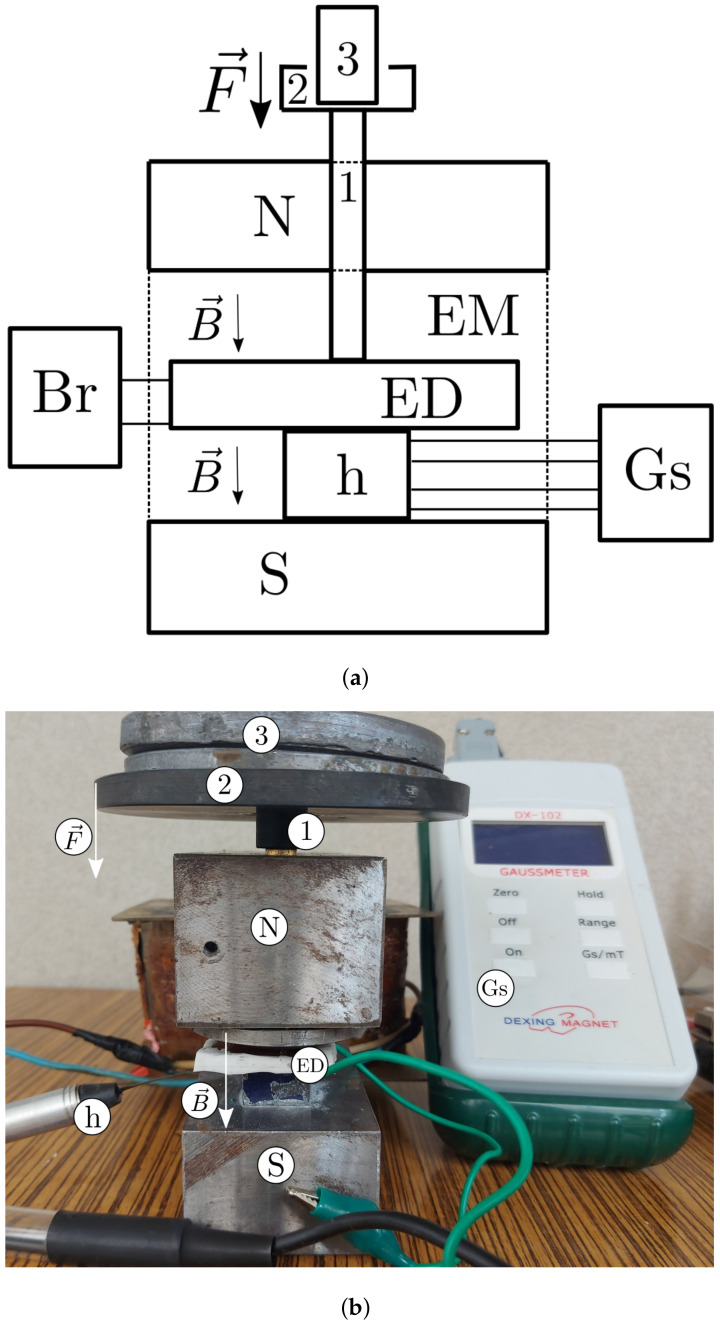
Ensemble configuration of the experimental installation: (**a**) schematic; (**b**) picture, featuring: EM, dc electromagnet with its N and S poles; Br, RLC bridge; Gs, gaussmeter; h, Hall Probe; $\vec{F}$, compression force vector; $\vec{B}$, magnetic density flux vector; 1, non-magnetic axle; 2, nonmagnetic plate/disc; 3, weight.

The experimental installation used for the study of the magnetocapacitive, magnetoresistive and magnetopiezoelectric effects of the ED devices had the overall configuration shown in Figure 11. The installation consisted of an electromagnet (EM), the dc source (DCS, not in the figure); a gaussmeter (Gs) with a Hall probe (h), a bridge (Br) and the computer (not in the figure), similar to the one used in [46], also used for measuring the response of electrical devices under similar conditions.

The electromagnet consisted of a magnetic U-shaped yoke and a coil. The magnetic yoke had a length $L_{j}=180\;mm$ and a width $l_{j}=50\;mm$. The magnetic poles of the electromagnet had a rectangular cross section of dimensions 50mm×40mm×mm. The distance between the magnetic poles was 50mm. In the north magnetic pole of the electromagnet, a hole of diameter 8mm was made and a brass shaft with a diameter of 7.8mm (position 1 in Figure 11) was inserted through the north pole of the electromagnet. A nonmagnetic plate/disk of diameter 110mm was attached to the upper end of the shaft (see position 2 in Figure 11), and a nonmagnetic disk was attached to the upper end of shaft 1.

The electromagnet’s coil had a dc resistance of $R_{b}=6.40\;\Omega$ and inductance $L_{b}=0.247\;H$. The electrical connection of the coil with the terminals of the dc source was made using electrical conductors with a section of $4.50\;{mm}^{2}$. The dc power source was an RXN-3020D, manufactured by Electronics Co., Ltd. (Shenzhen, China), and the voltage at its source terminals can be adjusted continuously, up to a maximum of 30 V (dc). On a resistive load, the intensity of the electric current discharged by the dc source was 60 A (dc) maximum.

The Gs gauge with a Hall h probe was a DX-102 manufactured by Dexing Magnet. It allows the measurement of magnetic flux densities up to 1000mT, with an accuracy of ±1%. The bridge was an 8846 A from Fluke that allows the measurement of the voltage, intensity and frequency of the electric current with high precision. Through the IEEE-488 and RS-232 interfaces, the measured values were transmitted to the computing unit, which was an Inspiron Core i7 laptop, equipped with software for the RS-232 interface and with experimental data processing software.

The experimental installation was configured as shown in Figure 11. The electrical devices (EDs) were inserted, one by one, between the disk and the Hall probe of the gaussmeter. Next, on the nonmagnetic plate of the installation, a nonmagnetic body was fixed, having a mass of 0.732kg. By pressing, a compression strain of τ≈7.98kPa was induced. Under this action, the ED device and the Hall probe of the Gs gauge were well fixed. It was estimated that, under the action of τ, the electrical contact resistance between the copper electrodes and the surfaces of the MAC composites was smaller by at least an order of magnitude compared to the intrinsic resistance *R* of the MAC samples.

At the start of each measurement cycle, the value *B* of the magnetic flux density was brought to a null value, by corresponding adjustments of the value and the direction of the electric current through the coil of the electromagnet. Using the bridge with the D and A filters active, the equivalent electrical capacity *C*, the equivalent electrical resistance *R* and the voltage *U* at the terminals of the electrical devices were measured for each value *B* of the magnetic flux density at time intervals Δt=15 s from the application of the magnetic field. During the measurements, a deviation of ±5% from the fixed value *B* of the magnetic flux density was allowed in the measurements.

## 3. Theoretical Framework

### 3.1. Aggregate Formation and the Equation of Movement for the Magnetic Dipoles

For modeling purposes, the CI microparticles and nBT nanoparticles are assumed to be one-dimensional, with diameters of $d_{1}=5\;\mu m$ (for CI) and $d_{2}\leq 10\;nm$ (for nBT), and evenly distributed in the CF mesh.

**Figure 12:**
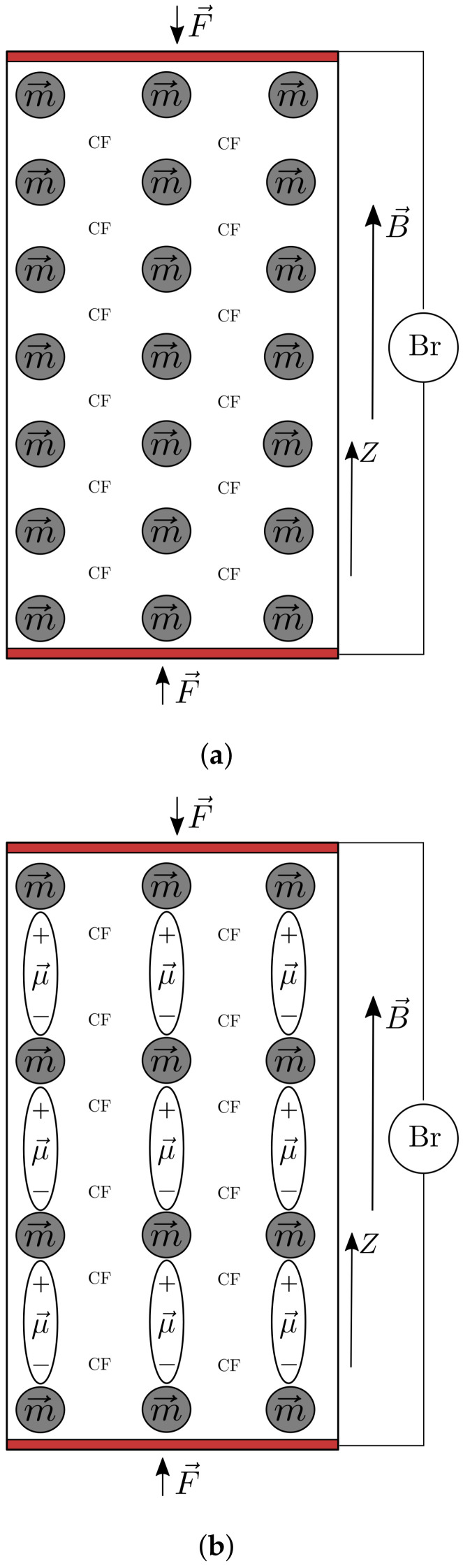
Model of the device: (**a**) in the absence of nBT and (**b**) in the presence of the nBT; $\vec{m}$, magnetic moment; $\vec{\mu }$, dipole electric moment; CF, cotton fiber fabric; $\vec{F}$, compression force; $\vec{B}$, magnetic flux density; Br, RLC bridge; *Z*, coordinate axis.

When placed in a magnetic field, the CI microparticles transform into magnetic dipoles. The moment $\vec{m}$ of the magnetic dipole, projected on the *Z* coordinate axis, as in Figure 12, is calculated with the expression [21,22,36]:

(1) $$m=\frac{\pi d_{1}^{3}B}{2\mu_{0}},$$

where: $d_{1}$ is the diameter of the magnetic dipole identical to that of the CI microparticles, *B* is the magnetic flux density and $\mu_{0}$ the magnetic permittivity of the vacuum.

The magnetic field in the volume of the ED capacitors is considered constant and homogeneous. Thus, the intensity of the magnetic interaction between two identical and neighboring magnetic dipoles, projected on the *z* axis is [21,22]:

(2) $$F_{mz}=-\frac{3\mu_{s}\mu_{0}m^{2}}{4\pi z^{4}},$$

where $\mu_{s}$ is the relative magnetic permeability of the CF tissue, $\mu_{0}$ is the magnetic permittivity of the vacuum, *m* is the dipole magnetic moment and *z* is the distance between the centers of mass of the magnetic dipoles at a certain time t>0. The minus sign indicates that the dipoles $\vec{m}$ are attracted to one another, as in Figure 12.

Under the action of the force $F_{mz}$, no matter how small, the assembly formed by the CI microparticles, electrostatically connected with the nBT nanoparticles and mixed with the air molecules, flows between the nBT nanoparticles and the CF fabric fibers. Therefore, in this sense, the mixture of CI microparticles, nBT nanoparticles and air molecules can be assimilated to a fluid. We consider that the polyphasic fluid thus formed had a dynamic viscosity. Then, the motion of the dipoles $\vec{m}$ is opposed by a resistance force, $F_{rz}$, which acts in the direction of the *z* axis, but in the opposite direction to the force $F_{mz}$. Moreover, we consider that the resistance force was of Stokes type, that is:

(3) $$F_{rz}=-3\pi \eta d_{1}\frac{dz}{dt},$$

where η is the dynamic viscosity of the fluid, $d_{1}$ is the magnetic dipole diameter, dz is the infinitesimal distance traveled by the dipoles $\vec{m}$ in the time interval dt.

Under the action of the forces $F_{mz}$ and $F_{rz}$, the dipoles $\vec{m}$ of mass *M* would receive an acceleration on the *z* axis, such that the second law of mechanics can be written as:

(4) $$M\frac{d^{2}z}{dt^{2}}=F_{mz}-F_{rz}.$$

By using the expressions of the forces from relations (2) and (3), the equation of movement for the dipoles $\vec{m}$ can be obtained:

(5) $$M\frac{d^{2}z}{dt^{2}}+\xi \frac{dz}{dt}+\frac{3\mu_{s}\mu_{0}m^{2}}{4\pi z^{4}}=0,$$

where ξ is the friction coefficient of the particles in the polyphasic fluid.

(6) $$\xi =3\pi \eta d_{1}.$$

and the viscosity can be expressed as [47]:

(7) $$\eta =\eta_{a}\eta_{rel},$$

where $\eta_{a}$ the viscosity of air at $25{\;}^{{}^{\circ}}$C and standard pressure, $\eta_{a}=1.8444\times 10^{-5}\;Pa$·s and $\eta_{rel}$ is the relative viscosity that can be calculated as a function of the volume fraction of CI microparticles, $\Phi_{\mathrm{CI}}$, with the empirical formula [48]:

(8) $$\eta_{rel}=\frac{1+0.5\Phi_{\mathrm{CI}}}{{\left(1-\Phi_{\mathrm{CI}}\right)}^{4}}.$$

For the values of $\Phi_{\mathrm{CI}}$ in the experiment and $d_{1}=5\;\mu m$, the parameters $\eta_{rel}$, η and ξ are calculated in Table 2. It can be observed that by decreasing the fraction of CI microparticles (which coincides with the increase in nBT nanoparticles), the viscosity η of the polyphasic fluid is increased.

For values of $\mu_{s}\approx 1$, $\mu_{0}=4\pi \times 10^{7}\;H/m$ and $z=d_{1}=5\;\mu m$, the dependence of the magnetic force has the value:

(9) $$F_{mz}=\frac{31}{16}\;\times \;10^{-5}\cdot B^{2}\left(mT\right),$$

the square dependence of the magnetic flux intensity showing that the force between two neighboring magnetic dipoles is sensibly influenced by the external magnetic field.

The mass of the dipole can be expressed using the mass density of the CI, $\rho_{1}=7860\;\;{kg/m}^{3}$, and the particle diameter $d_{1}=5\;\mu m$ as:

(10) $$M=\frac{\pi }{6}\rho_{1}d_{1}^{3}\approx 5.14\times 10^{-17}\;kg,$$

and because it has a relatively low value, one can neglect the contribution of the term containing it in (5), so that the movement equation can be written in a simplified form as:

(11) $$\frac{dz}{dt}+\frac{\mu_{s}d_{1}^{5}B^{2}}{4\mu_{0}\eta z^{4}}=0.$$

which can be solved for *z*. Denoting the separation of the dipoles δ=z(t), this quantity can be written as a function of the initial separation $\delta_{0}=z\left(0\right)$:

(12) $$\delta =\delta_{0}{\left[1-\frac{5\mu_{s}}{4\mu_{0}}{\left(\frac{d_{1}}{\delta_{0}}\right)}^{5}\frac{B^{2}t}{\eta }\right]}^{1/5}.$$

It can be observed that the distance between the centers of mass of the dipoles $\vec{m}$, similar to composite materials studied in [21,22,23,24,25,26,36,37], is dependent on the composition of the MACs through the quantities $\delta_{0}$, $d_{1}$ and η. The quantity δ sensibly decreases with the increase of the magnetic flux density *B*, while for a fixed composition of the MAC and a fixed value of *B*, δ decreases linearly with the increase of the time *t* it takes maintaining the composites in a magnetic field.

For brevity, we denote the parameter β containing the linear time dependence as:

(13) $$\beta =\frac{5\mu_{s}}{4\mu_{0}}{\left(\frac{d_{1}}{\delta_{0}}\right)}^{5}\cdot t,$$

and the parameter *K* that also contains the squared dependence on the magnetic field density as:

(14) $$K=1-\frac{\beta }{\eta }\cdot B^{2},$$

such that the parameter δ can be written simply as:

(15) $$\delta =\delta_{0}K^{1/5}.$$

### 3.2. Electrical Equivalent Components of the EDs

The number *N* of CI microparticles in the MACs can be estimated by:

(16) $$N=\frac{\Phi_{\mathrm{CI}}V}{V_{p}}=\frac{6\Phi_{\mathrm{CI}}Llh_{0}}{\pi d_{1}^{3}},$$

where $\Phi_{\mathrm{CI}}$ is the volume fraction of the CI microparticles, $V_{p}$ is the volume of a microparticle of diameter $d_{1}$, and *L*, *l* and $h_{0}$ are the dimensions of the MAC (length, width and thickness).

In a magnetic field, the CI microparticles are transformed into the dipoles $\vec{m}$ (Figure 12), which are oriented along the magnetic field’s lines. The maximum number of dipoles in a chain can be estimated with:

(17) $$N_{1}=\frac{h_{0}}{d_{1}},$$

where $h_{0}$ is the thickness of the MAC, equal to the distance between the electrodes of the ED, and $d_{1}$ is the diameter of the CI microparticles and, by extension, of the dipoles.

The chains of magnetic dipoles form a spatial network inside the MAC based on cotton fibers and CI microparticles, and a spatial aggregates network inside the one with added nBT nanoparticles (Figure 12).

The number $N_{2}$ of chains that form in the MAC can be approximated with:

(18) $$N_{2}=\frac{N}{N_{1}}=\frac{6\Phi_{\mathrm{CI}}Ll}{\pi d_{1}^{2}}.$$

In each chain, the common surface of two dipoles outlines a composite body that can be considered equivalent to an electric circuit formed from a plane microcapacitor $C_{z_{1}}$ connected in parallel to a linear microresistor $R_{z_{1}}$, whose capacitance and resistance can be calculated simply as:

(19) $$C_{z_{1}}=\varepsilon_{0}\varepsilon_{r}\frac{S}{\delta },$$

and

(20) $$R_{z_{1}}=\rho \frac{\delta }{S},$$

where $\varepsilon_{0}$ is the dielectric permittivity of the vacuum, $\varepsilon_{r}$ the relative dielectric permittivity, δ the distance between the centers of mass of the dipoles and *S* the surface area common to two dipoles, which can be calculated as:

(21) $$S=\frac{\pi d_{1}^{2}}{2}.$$

**Figure 13:**
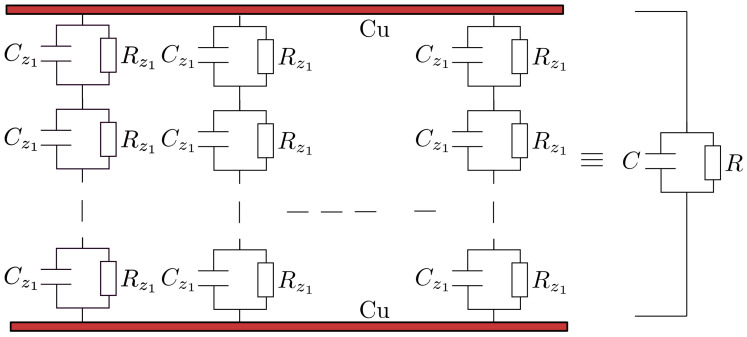
Equivalent electrical representation of ED devices: $C_{z_{1}}$, microcapacitor; $R_{z_{1}}$, microresistor; *C*, equivalent electrical capacity; *R*, equivalent electrical resistance; Cu, copper electrodes.

In each chain, the microcapacitors $C_{z_{1}}$, in parallel to the microresistors $R_{z_{1}}$ form a spatial electrical network, whose longitudinal section looks like the one presented in Figure 13.

Then, for $N_{1}>>1$, the equivalent electrical capacity of a chain is $C_{z_{c}}\approx \frac{C_{z_{1}}}{N_{1}}$, while the equivalent electrical resistance of a chain is $R_{z_{c}}\approx N_{1}R_{z_{1}}$. Taking into account that the dipole chains are connected in parallel between the copper electrodes of the EDs, we can calculate the equivalent capacity as $C=N_{2}C_{z_{c}}=\frac{N_{2}}{N_{1}}C_{z_{1}}$ and the equivalent electrical resistance as $R=\frac{R_{z_{C}}}{N_{2}}R_{z_{1}}=\frac{N_{1}}{N_{2}}R_{z_{1}}$ and, subsequently, taking into account the expressions for *N*, $N_{1}$, $N_{2}$ and *S*, we obtain:

(22) $$C=\frac{3\varepsilon_{0}\varepsilon_{r}\Phi_{\mathrm{CI}}d_{1}Ll}{h_{0}\delta },$$

and

(23) $$R=\frac{\rho h_{0}\delta }{3\Phi_{\mathrm{CI}}d_{1}Ll}.$$

By observing that $\delta =\delta_{0}K^{1/5}$, this also means that the values of *C* and *R* inherit the time dependence of δ, and can be written compactly as:

(24) $$C_{i}=C_{0i}K^{-1/5},$$

and

(25) $$R_{i}=R_{0i}K^{1/5}.$$

### 3.3. Voltage at Terminals and Equivalent Scheme of the EDs

It is known [49,50] that the nBT nanoparticles, under external mechanical action, spontaneously polarize. The polarization of the electric charges is determined by the direction of the application of the mechanical tension, as shown in Figure 12. Here, in the direction of the force $\vec{F}$, the nBT nanoparticles become electrically charged, the conversion of mechanical energy into electricity taking place [49], an effect known as piezoelectricity [50]. In the direction of the force $\vec{F}$, the nBT nanoparticles are charged with electrical charges (denoted by *q*), while on the armatures of the electrical devices ED, a potential difference *U* appears, which is measured by the dc millivoltmeter of the bridge. For a certain value *F* of the compression force, the value of the electric charge *q* is a constant quantity. On the other hand, since it is known that a body charged with an electric charge *q* has a potential difference *U* with respect to another body, and the connection is given by the quantity called electrical capacity *C*, one can write q=CU, so the potential difference inherits the time dependence as:

(26) $$U_{i}=U_{0i}K^{1/5},$$

where $U_{0}$ is

(27) $$U_{0}=\frac{qh_{0}\delta_{0}}{3\varepsilon_{0}\varepsilon_{r}\Phi_{\mathrm{CI}}d_{1}Ll}.$$

From the dependence of *U*, it can be observed that the difference of potential between the two terminals sensibly decreases with *B*, and it is influenced by the nBT volume fraction $\Phi_{\mathrm{nBT}}$, which directly affects the quantities η and $\delta_{0}$. Moreover, the initial difference of potential $U_{0}$ increases along with the quantities *q*, $h_{0}$ and $\delta_{0}$, but decreases with the volume fraction of CI microparticles $\Phi_{\mathrm{CI}}$, which happens alongside an increase of $\Phi_{\mathrm{nBT}}$.

**Figure 14:**
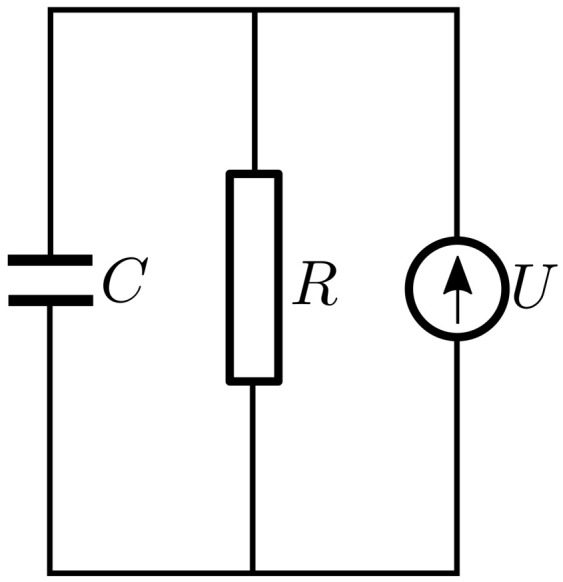
Equivalent electrical circuit of the EDs: *C*, capacitor; R, resistor; U, voltage source.

These considerations lead us to conclude that the electrical schematic of the ED devices based on MACs is the one shown in Figure 14—consisting of a capacitor connected in parallel with a resistor and a potential source.

The obtained results also suggest that, in a magnetic field, one can fix the values of the equivalent electric capacity *C*, of the equivalent resistance *R* and of the difference of potential *U* such that the assembled EDs have either capacitive, resistive, piezoelectric or a mixed character, as needed in applications.

## 4. Measurements and Discussion

**Figure 15:**
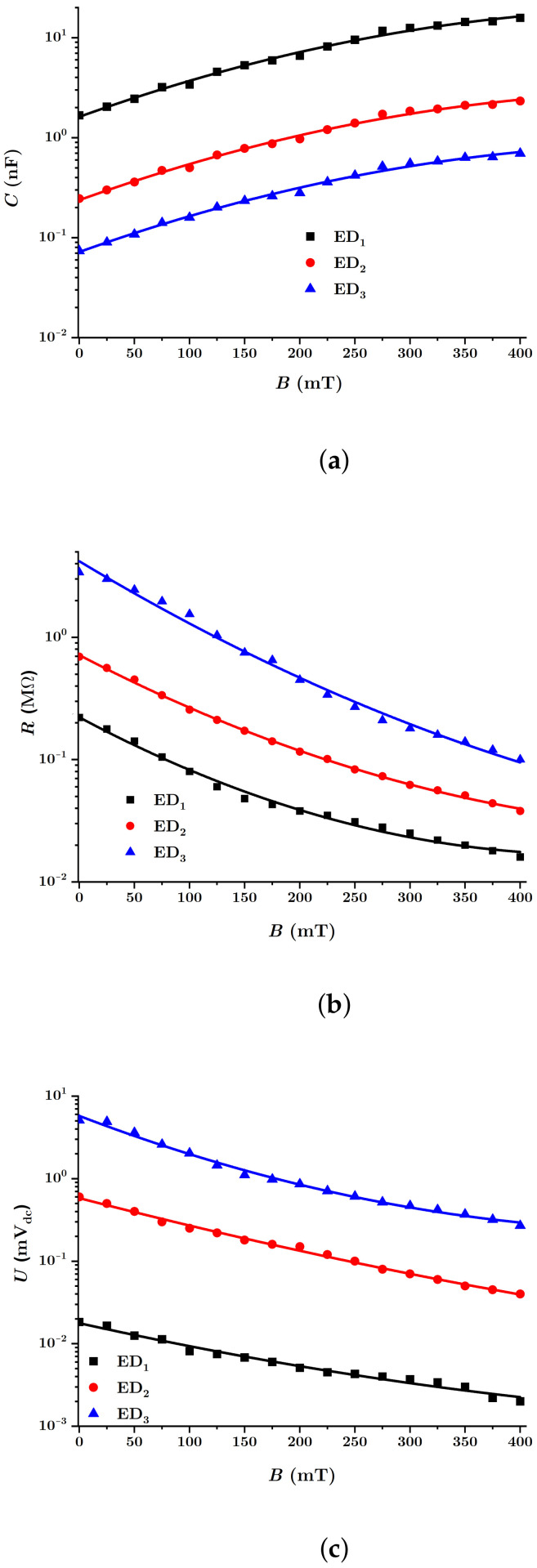
(**a**) Equivalent electrical capacity C; (**b**) equivalent electrical resistance R; (**c**) electrical voltage U at the terminals of the EDs as a function of values of the magnetic flux density *B* (points: experimental data, solid lines: theoretical data).

In Figure 15, the equivalent electrical capacities $C_{i}$, the equivalent electrical resistances $R_{i}$ and the values $U_{i}$ (with i=1,2,3) were measured at the terminals of the EDs, as a function of the values of the magnetic flux density *B*. The functions are denoted by $C_{i}={C_{i}\left(B\right)}_{{\mathrm{MAC}}_{i}}$, $R_{i}={R_{i}\left(B\right)}_{{\mathrm{MAC}}_{i}}$ and $U_{i}={U_{i}\left(B\right)}_{{\mathrm{MAC}}_{i}}$, with i=1,2,3.

It is observed from Figure 15a that the functions $C_{i}\left(B\right)$ have a rate that increases with the value of the magnetic flux density *B*. For fixed values of this quantity, the value $C_{i}$ of the equivalent electrical capacity decreases as the volume fraction $\Phi_{\mathrm{nBT}}$ of the barium titanate nanoparticles increases.

Increasing the value of the magnetic flux density has the effect of decreasing the distance δ between the centers of mass of the magnetic dipoles, as can be seen from Equation (15). The effect of the decrease of δ, with the increase of *B* gives the increase of the equivalent electrical capacities $C_{z_{1}}$ of the electric microcapacitors from Figure 13, and on the whole, the increase of the equivalent electrical capacity of the EDs with the increase of *B*, as can be seen in Figure 15a.

Figure 15b shows that the equivalent electrical resistance $R_{i}\left(B\right)$ of the EDs decreases significantly with the rise of the value of the magnetic flux density *B*. On the other hand, the values of $R_{i}$, for the same values *B* of the magnetic flux density, rise with the increase of the volume fraction $\Phi_{\mathrm{nBT}}$ of the barium titanate nanoparticles. Furthermore, in the absence of the magnetic field, the equivalent electrical resistances $R_{0i}$ of the EDs increase with the volume fraction $\Phi_{\mathrm{nBT}}$.

By increasing the magnetic flux density *B*, the distance δ between the centers of mass of the magnetic dipoles decreases according to Equation (15). Consequently, the equivalent electrical microresistors $R_{z_{1}}$ from Figure 13 are reduced and, on the whole, the equivalent electrical resistance of the EDs increases with the rise of the magnetic flux density, in accordance with Equation (25). On the other hand, the increase of the values $\Phi_{\mathrm{nBT}}$ in the MACs has the effect of increasing the initial distance between the centers of mass of the CI particles, in the absence of the magnetic field, which in turn affects the increase of the values of the equivalent electrical resistance of the EDs, as can be seen in Figure 15b.

Finally, it is observed from Figure 15c that the values $U_{i}\left(B\right)$ of the electric voltage measured at the terminals of the EDs are also influenced by the values of the magnetic flux density *B* and by the values of the volume fraction $\Phi_{\mathrm{nBT}}$ of the barium titanate nanoparticles. In the absence of the magnetic field, it is observed that the values $U_{0i}$ of the electrical voltage at the terminals of the electrical devices depend on the composition of the MAC.

Indeed, by increasing the magnetic flux density *B*, the voltage *U* at the terminals of the EDs [51] increases as an effect of magnetoconstriction. In contrast, in the absence of the magnetic field, the voltage $U_{0}$ increases along with the decrease of the volume fraction of CI microparticles and the increase of the initial distance $\delta_{0}$ and with the amount of nBT nanoparticles in the MACs, in accordance with Equation (26) and the experimental results from Figure 15c.

In [25], composites were fabricated from CI microparticles, γ-${Fe}_{2}O_{3}$ nanoparticles, silicone oil and cotton fibers, and the quantity $\delta_{0}$ was calculated with a certain relation, which, if considered with the nBT nanoparticles as fillers (instead of γ-${Fe}_{2}O_{3}$ nanoparticles), would read:

(28) $$\delta_{0}=\frac{1+d_{1}\frac{\Phi_{\mathrm{nBT}}}{\Phi_{\mathrm{CI}}}}{\sqrt[{3}]{\left(1+\Phi_{\mathrm{nBT}}\right)\Phi_{\mathrm{CI}}}},$$

where $d_{1}$ is the diameter of the CI microparticle, $\Phi_{\mathrm{nBT}}$ is the volume fraction of the additive (barium titanate nanoparticles) and $\Phi_{\mathrm{CI}}$ is the volume fraction of the CI microparticles.

Then, in Table 1, values for the $\delta_{0}$ parameter were obtained for $d_{1}=5\;\mu m$, and the values for $\Phi_{\mathrm{nBT}}$ and $\Phi_{\mathrm{CI}}$ from Table 3, introduced in Equation (28).

It can be seen from Table 3 that the initial distance between the centers of mass of the dipoles $\vec{m}$ increases with the amount of nBT nanoparticles used.

In the expression for the parameter β (13), by setting the values for $\mu_{s}\approx 1$, $\mu_{0}=4\pi \times 10^{-7}\;H/m$, $d_{1}=5\;\mu m$, t=Δt=15s and $\delta_{0i}$ (with i=1,2,3) values from Table 3, values for $\beta_{i}$ were obtained. It can be observed that these values decrease as the ratio of volume fractions $\Phi_{\mathrm{nBT}}$ and $\Phi_{\mathrm{CI}}$ increases.

It is known [25] that in composites based on CI microparticles, silicone oil and γ-${Fe}_{2}O_{3}$ nanoparticles, the viscosity of the composites changes the magnetic field according to a law of the form:

(29) $$\eta_{i}=\eta_{0i}-\alpha_{i1}\cdot B+\alpha_{i2}\cdot B^{2},$$

To determine the values of Function (29) we use Equations (13), (14) and (24) from which we obtain:

(30) $$\eta_{i}=\beta_{i}\frac{B^{2}}{1-{\left(\frac{C_{0i}}{C_{i}}\right)}^{5}},$$

**Figure 16:**
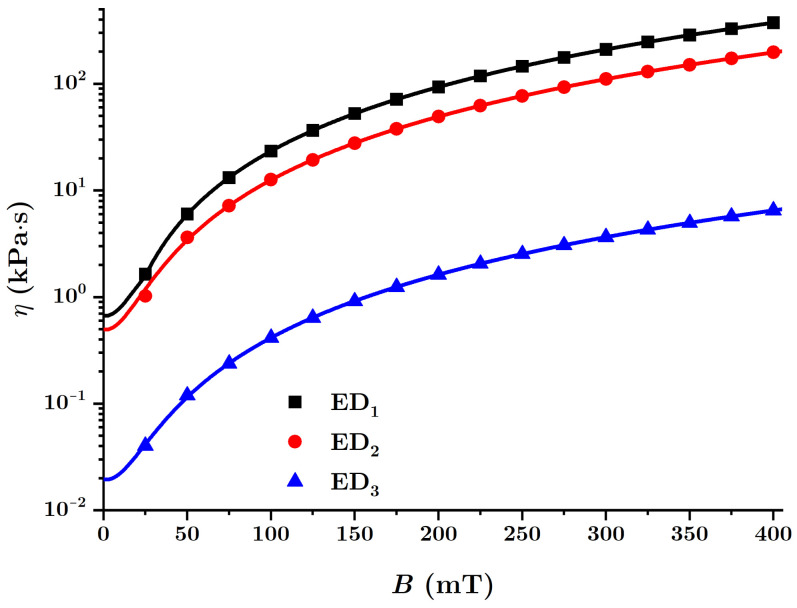
The viscosity η of the MAC composites within the EDs as a function of the magnetic flux density *B*.

For the quantities $\beta_{i}$, with i=1,2,3, in Table 3, the functions $C_{i}$ as determined in Figure 15a and values of the magnetic field density between 0 and 400mT as introduced in Equation (30), the dependence $\eta_{i}=\eta_{i}{\left(B\right)}_{{\mathrm{MAC}}_{i}}$ was obtained for the three EDs, as shown in Figure 16.

From Figure 16, it is observed that the viscosity increases significantly as the value of the magnetic flux density *B* increases. However, by introducing nBT nanoparticles, the intensity of the magnetic interaction between the dipoles $\vec{m}$ decreases and the effect obtained is a decrease of the viscosity η, as shown in Figure 16. The functions $\eta_{i}{\left(B\right)}_{{\mathrm{MAC}}_{i}}$ for the three EDs illustrated in Figure 16 have the form given in Equation (29), the parameters $\eta_{0i}$, $\alpha_{i1}$ and $\alpha_{i2}$ being the ones given in Table 3.

Using the results from Table 3, the shapes of the functions $C_{i}\left(B\right)$, $R_{i}\left(B\right)$ and $U_{i}\left(B\right)$ were obtained, which fitted the experimental data from Figure 15c.

Furthermore, it is of note that for the values $\eta_{0i}$ of the dynamic viscosity, shown in Table 3, the reduction of the equation of motion of the dipoles $\vec{m}$ from Equation (5) to (11) is justified.

We define the magnetocapacitive ($\mu_{C}$), the magnetoresistive ($\mu_{R}$) and the magnetopiezoelectric ($\mu_{U}$) effects by the relations:

(31) $$\mu_{C}=\frac{C-C_{0}}{B},$$

(32) $$\mu_{R}=\frac{R_{0}-R}{B},$$

(33) $$\mu_{U}=\frac{U_{0}-U}{B},$$

which quantify the quality of the EDs fabricated using the MACs for an arbitrary value of the magnetic flux density *B*, where $C_{0}$, $R_{0}$ and $U_{0}$ are, respectively, the equivalent electrical capacities, resistances and potential differences measured at the terminals of each ED in the absence of a magnetic field.

Using Equations (24)–(26), we obtain:

(34) $$\mu_{\mathrm{CI}}=\frac{C_{0i}}{B}\left(K_{i}^{-1/5}-1\right),$$

(35) $$\mu_{Ri}=\frac{R_{0i}}{B}\left(1-K_{i}^{1/5}\right),$$

(36) $$\mu_{Ui}=\frac{U_{0i}}{B}\left(1-K_{i}^{1/5}\right).$$

**Figure 17:**
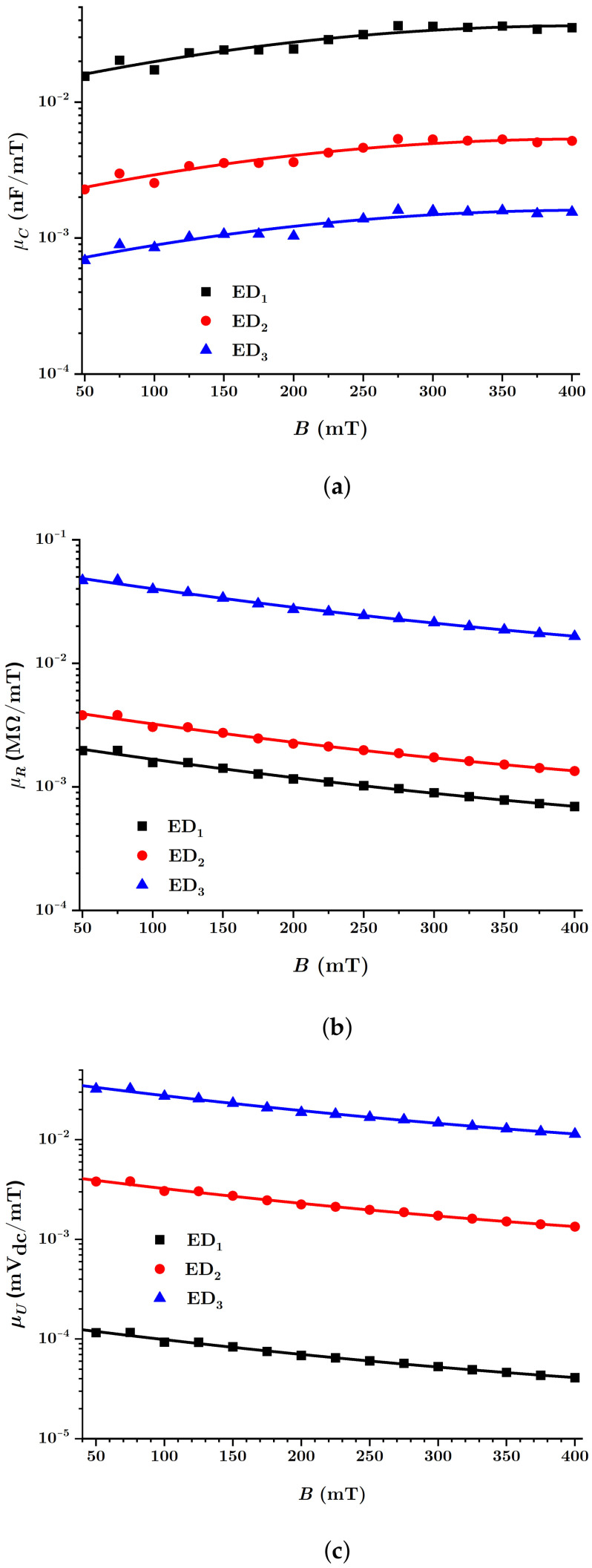
(**a**) Magnetocapacitive $\mu_{C}$, (**b**) magnetoresistive $\mu_{R}$ and (**c**) magnetopiezoelectric $\mu_{U}$ effects of electrical devices made with MACs, as a function of values of the magnetic flux density *B* (points = experimental data, solid lines = theoretical data).

These new functions can be plotted using the experimental data from Figure 15, resulting in Figure 17.

As expected, the quantities $\mu_{C}$, $\mu_{R}$ and $\mu_{U}$ are significantly influenced by both the composition of the MACs and the values of the magnetic flux density. Regarding the response functions of the ED devices to magnetic excitations, with reference to Figure 17, we note the following: while the quantity $\mu_{C}$ decreases, $\mu_{R}$ and $\mu_{U}$ increase, for the same values of *B*, when the volume fraction $\Phi_{\mathrm{nBT}}$ of barium titanate nanoparticles increases.

The MAC compression tension between the copper electrodes of the EDs and the local magnetoconstriction caused by the magnetic field results in the appearance of electric charges (see Figure 12). The electric charge *q* accumulated on the electrodes of the ED devices is obtained simply as q=CU, where *C* is the equivalent electric capacitance of the device and *U* the potential difference at the terminals of the ED.

**Figure 18:**
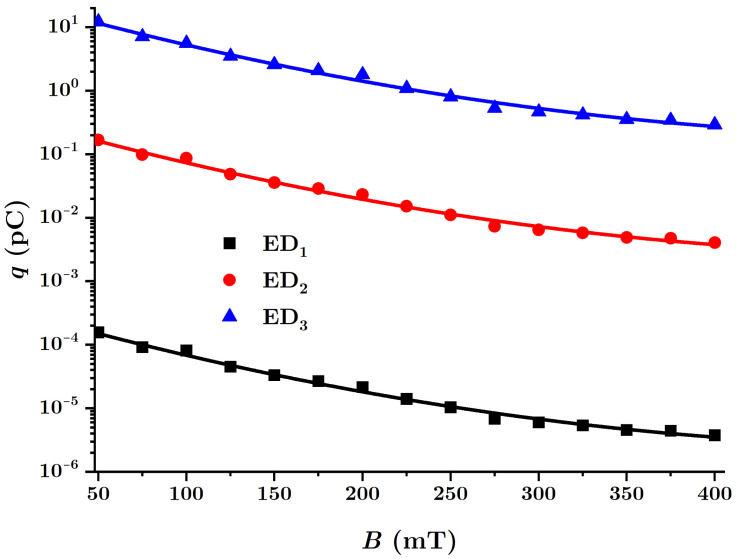
The amount of electrical charge *q* accumulated on the electrode surfaces of the EDs as a function of the magnetic flux density *B*.

By using the functions $C_{i}\left(B\right)$ and $U_{i}\left(B\right)$, one can obtain this charge for the three devices as:

(37) $$q_{i}\left(B\right)=C_{i}\left(B\right)U_{i}\left(B\right),$$

which is plotted in Figure 18.

It is observed from Figure 18 that, on the surface of the electrodes of the ED devices, the electric charge *q* increases with the increase of the quantity of nBT nanoparticles. The growth is an order of magnitude greater than in the case without nBT nanoparticles.

Indeed [49,50,52], by increasing the number of nBT nanoparticles, for the same value of the compression stress τ, there is an increase in the amount *q* of electrical charge. In a magnetic field, the magnetic force $F_{mz}$ acts on the dipoles $\vec{m}$. In addition to the electrification generated by friction between the dipoles and the fabric, and the increase in the local compression caused by the rise in the magnetic force, the obtained effect is the change in the amount of electric charge on the surface of the electrodes. Thus, by accumulation, one can obtain maximum values for the electric charges (Figure 18), values influenced by the composition of the MACs and the magnetic flux density *B*. On the other hand, increasing the value of *B* decreases the internal resistance *R* (see Figure 15b), and the obtained effect consists of an increase in the intensity of the electric leakage current, along with a decrease in the amount of electrical charge *q* accumulated on the surface of the ED electrodes (Figure 18).

**Figure 19:**
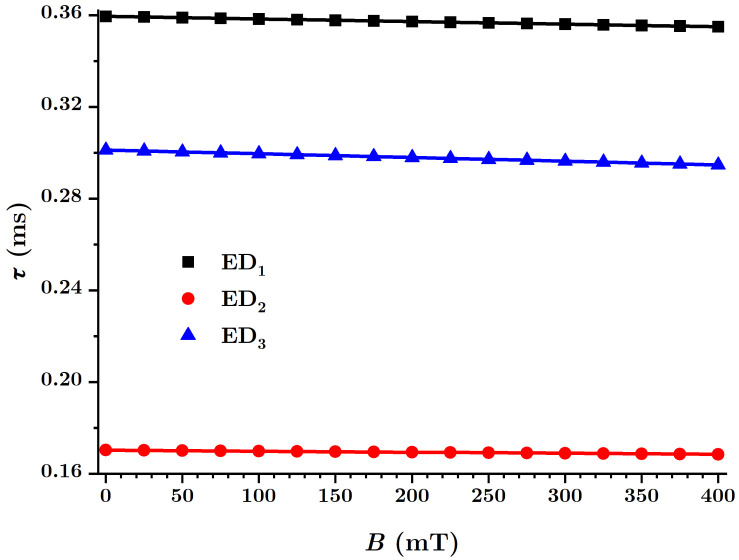
The time constant τ of the EDs as a function of the magnetic flux density *B*.

The time constant of the EDs is defined by the relation:

(38) τ(ms)=R(MΩ)·C(nF),

in which *R* is the electrical resistance and *C* is the equivalent electrical capacity of the EDs. By using the data from Figure 15, the quantities $\tau_{i}=\tau_{i}{\left(B\right)}_{{\mathrm{ED}}_{i}}$ are obtained, as shown in Figure 19.

From Figure 19 it can be seen that the functions $\tau_{i}$ are linear in *B*, thus varying with the magnetic flux density as:

(39) $$\tau_{i}=a_{i}+b_{i}T,$$

where the coefficients $a_{i}$ and $b_{i}$ are given in Table 4. It is observed from Figure 19 that the time τ, in the absence of the magnetic field, decreases with the amount of nBT nanoparticles, and that all three EDs show a slow, linear decrease with the magnetic flux density *B*.

## 5. Conclusions

Magnetically active composite membranes are fabricated by doping cotton fabrics with carbonyl iron microparticles and barium titanate nanoparticles. The electrostatic loading of the cotton fabric and barium titanate nanoparticles makes the particles well-attached to the fabric, as can be seen from SEM analyses (Figure 7a–c). Electric devices were made out of magnetically active compounds using copper and silicone rubber fittings (Figure 10b). In magnetic fields, the magnetoconstriction of the composites took place. The observed effects consisted of the increase of the electrical capacity (Figure 15a), the decrease of the electrical resistance (Figure 15b) and the increase of the electrical voltage (Figure 15c) at the devices’ terminals when the magnetic flux density was increased, according to the elaborated model: Equations (24) and (22) for the electrical capacitance, Equations (25) and (23) for the electrical resistance and Equations (26) and (27) for the potential difference. It was shown that the magnetocapacitive effects decreased (Figure 17a), while, on the other hand, the magnetoresistive effects (Figure 17b) and the magnetopiezoelectric ones increased (Figure 17c) with the magnetic flux density. Moreover, the time constant (Figure 19) of the electrical devices had a slow linear variation with the magnetic flux density and was influenced by the amount of nanoparticles used. These obtained effects can be useful in the realization of electrical devices for sensing mechanical vibrations or shocks and in transducers for mechanical deformations and tensions.

## Figures and Tables

**Table 1:** Components of the MACs.

Sample	$V_{\mathrm{CI}}\;\left({\mathrm{cm}}^{3}\right)$	$V_{\mathrm{nBT}}\;\left({\mathrm{cm}}^{3}\right)$	$V_{f}\;\left({\mathrm{cm}}^{3}\right)$	$\Phi_{\mathrm{CI}}\;\left(\%\right)$	$\Phi_{\mathrm{nBT}}\;\left(\%\right)$	$\Phi_{\mathrm{CF}}\;\left(\%\right)$	$h_{0}\;\left(\mathrm{mm}\right)$
MAC${}_{1}$	0.10	0.00	0.485	17.0	0.0	83.0	0.65
MAC${}_{2}$	0.10	0.10	0.485	14.6	14.6	70.8	0.76
MAC${}_{3}$	0.10	0.20	0.485	12.7	25.4	61.9	0.87

**Table 2:** Viscosity and friction parameters for the MACs.

Sample	$\Phi_{\mathrm{CI}}$	$\eta_{\mathrm{rel}}$	$\eta \;(10^{-5}\;\mathrm{Pa}$·s)	$\xi \;(10^{-9}\;\mathrm{kg}/s)$
MAC${}_{1}$	0.170	2.1450	3.9563	1.8643
MAC${}_{2}$	0.146	2.0173	3.7207	1.7533
MAC${}_{3}$	0.127	1.8309	3.3770	1.5914

**Table 3:** Characteristic quantities of the MACs in the ED devices.

Sample	$\delta_{0}\;\left(\mu m\right)$	$\eta_{0}\;(\mathrm{kPa}$·s)	$\alpha_{i1}\;(\mathrm{kPa}$·${s/\mathrm{mT}}^{2})$	$\alpha_{i2}\;(\mathrm{kPa}$·${s/\mathrm{mT}}^{2})$	β(kPa·${s/\mathrm{mT}}^{2})$
MAC${}_{1}$	9.0260	0.420	0.0015	0.002300	0.1950
MAC${}_{2}$	10.889	0.220	0.0031	0.001240	0.1110
MAC${}_{3}$	20.294	0.019	0.0013	0.000041	0.0012

**Table 4:** Coefficients in the linear dependence of the tie constant τ.

Sample	$a_{i}\;\left(\mathrm{ms}\right)$	$b_{i}\;\left(10^{-5}\;\mathrm{ms}/\mathrm{mT}\right)$
ED${}_{1}$	0.35948	−1.13419
ED${}_{2}$	0.17034	−0.45348
ED${}_{3}$	0.30122	−1.62285

## Data Availability

Not applicable.

## Abbreviations

The following abbreviations are used in this manuscript:

ML	Magnetic liquid
MRS	Magnetorheological suspension
MRE	Magnetorheological elastomer
MAC	Magnetically active composite
CI	Carbonyl iron
nBT	Barium titanate nanoparticles
CF	Cotton fiber
ED	Electrical device
SEM	Scanning electron microscope
EDX	Energy dispersive X-ray analysis
